# Steelhead trout (*Oncorhynchus mykiss*) fed probiotic during the earliest developmental stages have enhanced growth rates and intestinal microbiome bacterial diversity

**DOI:** 10.3389/fmars.2022.1021647

**Published:** 2022-11-14

**Authors:** Ian S. Hines, Kevin D. Santiago-Morales, Clay S. Ferguson, Jireh Clarington, Meaghan Thompson, Meghann Rauschenbach, Uri Levine, David Drahos, Frank O. Aylward, Stephen A. Smith, David D. Kuhn, Ann M. Stevens

**Affiliations:** 1 Department of Biological Sciences, Virginia Tech, Blacksburg, VA, United States; 2 Center for Emerging, Zoonotic, and Arthropod-borne Pathogens, Virginia Tech, Blacksburg, VA, United States; 3 Department of Food Science and Technology, Virginia Tech, Blacksburg, VA, United States; 4 Novozymes Biologicals Inc., Salem, VA, United States; 5 Department of Biomedical Sciences and Pathobiology, Virginia Tech, Blacksburg, VA, United States

**Keywords:** aquaculture, *Bacillus subtilis*, fish development, microbiome, *Oncorhynchus mykiss*

## Abstract

Sustainable aquaculture practices can help meet the increasing human demand for seafood, while easing pressures on natural fish populations. Studies aimed at increasing fish production in aquaculture have included supplementary dietary probiotics that often promote general health and enhanced growth rates by altering the microbiome of the host. Steelhead trout (*Oncorhynchus mykiss*) is anadromous, like salmon, and it is a subspecies of rainbow trout capable of rapid growth, making it an attractive fish to the aquaculture industry. In this study, the impact of feeding a *Bacillus subtilis* probiotic on the bacterial microbiome of steelhead trout was examined temporally across several stages of animal development, from eggs (day −19) through 184 days after hatching, in relation to physiological measures. Diets included: commercial feed only as a control (A), continually-fed probiotic top-coated on commercial feed (B), commercial then switch to probiotic feed (C), or probiotic then switch to commercial feed (D). Validation of probiotic concentrations on feed and in fish tissues was performed using CFU/g and qPCR, respectively. Fish growth was measured and samples for intestinal microbiome analyses were collected at multiple timepoints during fish development. Fish fed diet D yielded higher weights than the other three diets, with little impact on other biometric parameters. However, bacterial microbiome analysis indicated an increasing trend of overall alpha diversity from the egg stage to day 29 for fish fed the various diets with diet D having the highest diversity. Fish fed diets A and D maintained a high alpha diversity beyond day 29 in contrast to a decreased trend for fish still being fed probiotics in diets B and C. The fish fed diets B and C harbored a significantly higher relative abundance of *Bacillus* sp. in their total microbiomes (feces + mucosa). Interestingly, the mucosal-only microbiome indicated little variation between the four groups of fish. Feeding the probiotic earlier in development, during the hatchery phase, to influence bacterial microbiome composition in the intestine (rather than later after the microbiome has been established) appears to be a more effective aquaculture practice by enhancing microbiome diversity while enabling higher fish yields.

## Introduction

Fish are an important global agricultural resource, especially by providing a valuable source of animal protein in the human diet. However, wild-caught fishing practices are increasingly unable to meet the demands of a growing human population and this has led to over-exploitation of some fishing sources (FAO, 2020). The aquaculture industry has proved to be an effective alternative means of supplying fish products and it is now the fastest growing sector of food production, estimated to be over a $250 billion industry (FAO, 2020). With this increased reliance on aquaculture, sustainability is an important priority. Sustainability is dependent on animal welfare and health, which in turn are dependent on the associated microbiome.

Bacteria that inhabit the various external and internal environments of fish hosts constitute the fish bacterial microbiome. These microbial communities play vital roles in maintaining gastrointestinal homeostasis (Merrifield and Rodiles, 2015; Butt and Volkoff, 2019; Xu et al., 2020; Yu et al., 2021), protection against pathogens (Gomez et al., 2013; de Bruijn et al., 2018), and nutrient acquisition (Hanning and Diaz-Sanchez, 2015; Brugman et al., 2018). Exogenous agents, such as pre- and probiotics, can also increase the efficacy of these benefits (Kumar et al., 2008; Pirarat et al., 2011; Tuan et al., 2013; Sookchaiyaporn et al., 2020). Prebiotics are beneficial molecules processed through microbial metabolism, while probiotics are beneficial live microbial organisms. One such probiotic, *Bacillus subtilis*, the focus of this study, is capable of helping to increase fish growth rates (Park et al., 2017) while supporting the host immune system (Newaj-Fyzul et al., 2007; Kamgar et al., 2013; Standen et al., 2015; Galagarza et al., 2018; Tang et al., 2019) when is it added as a dietary supplement.

During fish intestinal development there exists a flux in the types and abundance of bacteria inhabiting the internal microbiome (Hansen and Olafsen, 1999). This flux is the result of the developing intestine being colonized by environmental microorganisms competing for space and the available nutrients within the gastrointestinal tract of the fish. Microbial communities within the developing fish intestine are strongly affected by diet and rearing conditions (Michl et al., 2017; Wilkes Walburn et al., 2019). There is some evidence that communities can be “seeded” *via* early dietary measures, with microbiome changes persisting into maturity (Parata et al., 2020 ). However, as the fish mature, the growing gastrointestinal microbiome will remain in flux, to some degree, as the host system continually selects for the most appropriate populations in the community (Ingerslev et al., 2014; Bakke et al., 2015; Stephens et al., 2016; Li et al., 2017). Therefore, it is vital that probiotics are administered in such a manner to facilitate effective colonization in the host. Unfortunately, exogenously-fed probiotics such as *B. subtilis* are typically unable to compete with an established native microbiome long-term to persist in the host tissues (Casula and Cutting, 2002; Giatsis et al., 2016) and must be supplied for the duration of fish culturing. Persistence of probiotic organisms in the microbiome of animals following probiotic cessation is an important attribute to consider during probiotic selection (Pérez-Sánchez et al., 2014).

For the present study, the impact of a probiotic on the earliest stages of fish development, when the microbiome is first developing once the animals hatch, versus later stages of growth, after an initial microbiome has been established, was examined. The salmonid, steelhead trout (*Oncorhynchus mykiss*), was chosen as the host animal for analysis, in part, due to its palatability and relatively understudied nature. Unlike rainbow trout (also *Oncorhynchus mykiss*), steelhead trout are anadromous and spend part of their life cycles in marine environments, but spawn in freshwater like salmon. It was hypothesized that exposure to probiotics at the early stages of intestinal development (i.e., first feeding) would lead to more effective intestinal colonization, and enhanced animal production. Overall, feeding the probiotic exclusively during early intestinal development led to the highest individual fish weights, while enabling greater bacterial microbiome diversity.

## Materials and methods

### Fish husbandry

Approximately 2000 steelhead trout eggs were supplied by Riverence Brood LLC (Olympia, WA, USA) and distributed evenly onto three vertical tray fish incubators (MariSource, Burlington, WA, USA). Fish incubators were situated on existing holding tanks as part of a single-system recirculating aquaculture system (RAS) using dechlorinated municipal water and dual 120 watt UV sterilizers. The RAS system was set up as described in Hines et al. (2021) with natural light (August – March) and fluorescent lighting on a 12h light:12h dark cycle. RAS water quality was monitored for temperature and dissolved oxygen on a daily basis, total ammonia-N and nitrite-N every other day, and nitrate-N and alkalinity on a weekly basis (AOCS, 2010; APHA, 2012). The water temperature was set to 11°C until eggs hatched at which point the temperature was set to 13°C for the duration of the study. Animals were maintained according to Virginia Tech IACUC #20–084.

**FIGURE 1:**
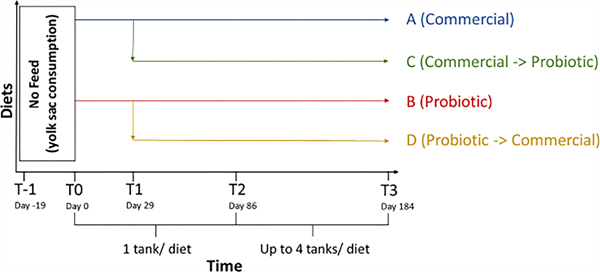
Timeline for steelhead trout harvests and feeding schedule. The water column and internal tissues of trout were collected at the indicated timepoints relative to the date of first exogenous feeding (T-1, Day −19; T0, Day 0; T1, Day 29; T2, Day 86; T3, Day 184). Fish were fed one of four diet regimens starting at T0 with the C and D diet groups differentiated at T1. The probiotic concentration for diets B, C, and D was ~10^8^ CFU/g.

Microbiome samples and fish weights were collected at multiple timepoints throughout the study (Figure 1). The first timepoint as defined by the receipt of the eggs was denoted as T-1 (day −19). Exogenous feeding was initialized 19 days after T-1 (T0; day 0) at which point the fish were equally distributed (~900 per tank) into two tanks and fed either a commercial feed control (A) or a probiotic-coated feed (B). The probiotic used in this study was *Bacillus subtilis* 086 spores (NZ86, Galagarza et al., 2018) supplied by Novozymes Biologicals Inc. (Salem, VA, USA) that was top-coated on to a commercial feed (Zeigler Bros. Inc, Gardners, PA, USA) at a concentration of 10^8^ CFU/g feed as confirmed using total aerobic plate counts in the appropriate medium after heating the feed to 80°C (killing the majority of non-spore bacteria) and activating the spores (Galagarza et al., 2018). Half of the fish from each tank (A and B treatment groups) were transferred into a new tank 29 days after first feeding (T1, day 29). At T1, 50% of the A treatment group continued on diet A, while the other half of the fish transferred from diet group A were switched to a probiotic diet (C). Similarly, 50% of the B treatment group continued on diet B, while the other half of the fish transferred from diet group B began a feeding regime using the commercial feed diet (D). To maintain appropriate production densities of fish, they began in non-replicated tanks and as they grew they were distributed to replicated tanks to increase scientific robustness. From T1 to T2 (day 86, ~450 fish per tank), one tank per dietary treatment was used. The production period (with tank replication) began at T2. At this time point, fish populations were reduced and equally distributed to 76 fish per tank to continue dietary treatments A through D with duplicate replication at the tank level. Fish were re-distributed two more times at day 115, increasing from two to three tanks per diet (~50 fish per tank), and again at day 128, increasing from three to four tanks per diet (~38 fish per tank). The study was completed with a final harvest at T3, day 184.

Each tank of fish was fed the same amount of feed to satiation during the early hatchery phase. The amount of feed used during the early hatchery phase was calculated by determining the mean weight of fish across all tanks. Fish were weighed bi-weekly on a per-tank basis starting at day 115. Tank densities (i.e., accounting for the total fish populations and average weights) were used as a basis to adjust the feeding regime to maintain adequate growth based on a dynamic percent body-weight feeding model additionally defined by the ambient water temperature, ~13°C (Hinshaw, 1999). Following each bi-weekly weighing, a bodyweight-based daily feeding model was adjusted to include the new data. The daily feeding rate decreased from 4.9% to 3.9% of bodyweight over the course of the production period as the trout grew. Additionally, the feed conversion ratio (FCR) was calculated using the bi-weekly growth and feed weight data. To indicate relative weight gains and control for fillet sampling bias, the Fulton’s condition factor (K) was employed (K = (weight(g)/full length (cm)^3^)*100, (Nash et al., 2006)).

### Sample acquisition strategy

Beginning at T-1 (receipt of eggs), 20 eggs were set aside on a sterile cheesecloth suspended over a beaker for microbiome sampling. Ten of these eggs were surface disinfected briefly with 1 mL 25 ppm iodine solution and rinsed with 2 mL of sterile phosphate buffered saline (PBS) for approximately 5 sec per egg; the remaining ten eggs were similarly rinsed only with sterile PBS. Each egg was individually homogenized for microbiome processing using pestles (Bel-Art, South Wayne, NJ, USA) surface-disinfected with 100% ethanol.

Immediately prior to initial exogenous feeding at T0 (day 0), ten fish were anesthetized in a 250 mg/L buffered MS-222 (Western Chemical Inc., Ferndale, WA) water bath, surface disinfected with 70% ethanol, and then rinsed for ~5 sec with 2 mL sterile PBS to reduce contamination from the skin microbiome. The heads and gills were removed from these fish prior to homogenization of the remaining tissues, including the intestines, with a hand-held tissue homogenizer (Bel-Art) for microbiome sampling. For time points T1, T2, and T3, fish were not fed the same day prior to being harvested in the morning (~18 hour feed restriction period prior to harvest). Then at T1 (day 29) ten fish each from diets A and B were processed in the same manner as the T0-processed samples described above. After euthanizing ten fish of each diet at T2 (day 86), the fish were now large enough that whole intestinal segments (with pyloric ceca and rectums removed, but including feces), were dissected out of each animal and subsequently homogenized for microbiome processing. Fish intestinal segments were homogenized using a surface-disinfected OmniTip homogenizer (Omni International, Kennesaw, GA, USA). A final harvest at T3 (day 184) included microbiome samples from diets A through D harvested in the same manner as T2. In addition, another ten intestinal segments per diet at T3 were extracted and manually cleared of fecal material by gentle squeezing then swabbed with sterile cotton-tipped swabs (Fisher brand, Pittsburgh, PA, USA) to obtain adherent microbiome samples (Reinhart et al., 2019; Clinton et al., 2021). Water column samples were also collected at the various timepoints by applying vacuum-filtration (Corning 0.22 μm filter, Corning, NY, USA) to 2.5 L of tank water. Sterile cotton swabs were used to collect the filter retentate.

Separately, 32 fish per diet (eight fish per tank from four tanks) were harvested at T3 and measured for the following biometrics: weight and length, viscerosomatic index (VSI), hepatosomatic index (HSI), fillet and ribless fillet yields, and muscle ratio for all diets A-D.

### Probiotic ingestion

Additional samples separately collected at T1 and T2 (processed in the same manner as the microbiome samples) and aliquots from the T3 microbiome homogenate samples were used for qPCR analysis of probiotic consumption. First, genomic DNA was isolated from the tissue homogenates using the Qiagen PowerLyzer PowerSoil kit and Qiagen Qiacube (Germantown, MD, USA). After slicing the homogenates into smaller pieces and bead beating using PowerBead tubes, 750 μL PowerBead solution was added. Following addition of solution C1, the PowerBead tubes were transferred onto the FastPrep system and set to shake at 1600 rpm for 1 min. Samples were then subjected to 13,000 x g centrifugation after which 450 μL of supernatant was transferred to a Qiacube cuvette. The Qiacube protocol was followed per the manufacturer’s procedure. Samples were then stored at −20°C until ready for qPCR-amplification.

Reaction concentrations for qPCR-amplification were as follows: 2 μL of the final 100 μL elution volume of gDNA isolated *via* the Qiacube protocol, 200 nM each of the forward (CTGTTCTCATGAACTGGGGC) and reverse (GCTAACTCTGCAGGTACCCC) primers targeting the *B. subtilis* strain 086, 100 nM of the probe ([FAM] AAGGTCGAAGTTGAGGCAAA[BHQ1a~6FAM]), 10 μL of LightCycler 480 Probes Master (Roche, Rotkreuz, Switzerland) and 5 μL of dH_2_O for a final volume of 20 μL. qPCR thermal cycler (Roche) settings included: initial denaturation at 95°C for 10 min, 36 cycles including denaturation at 95°C for 10 sec and annealing at 61°C for 30 sec, and a final cooling step at 37°C for 30 sec. At T1, 18 and 12 samples were taken from diet A and diet B fish, respectively. At timepoints T2 and T3, 18 samples were obtained from diet A fish and 12 samples from fish fed diets B, C, and D. Six additional samples were collected from fish fed diet A (no probiotic) than the other diets at each timepoint and artificially spiked with known concentrations of the *B. subtilis* 086 probiotic to create a standard curve prior to qPCR analysis so qPCR results could be correlated with CFU probiotic/gm fish homogenates. Probiotic detection in the water column was accomplished by taking 1 mL water samples from the system for analysis.

### DNA extractions for microbiome analysis

Genomic DNA (gDNA) was isolated from tissue homogenates and water column samples using the Qiagen PowerSoil kit per the manufacturer’s protocol with alterations including a 10-min incubation at 72°C after addition of C1 buffer and a 5-min incubation at 72°C prior to elution in 50 μL dH_2_O. Tissue homogenates were added to the PowerBead (Qiagen) tubes using a range of weights between 10 and 60 mg. Water column filter retentate swabs and T3 intestinal swabs were added directly to the PowerBead tubes. Prior to sample storage at −20°C, total gDNA quantity and purity (i.e., A_260_/A_280_ and A_260_/A_280_) were analyzed *via* a nanospectrophotometer (Implen, Westlake Village, CA).

### PCR amplification for microbiome analysis

The gDNA of tissue and water column samples was used as template to PCR-amplify the V4 region of the bacterial 16S rRNA gene (Table S1). Amplification reactions were done in triplicate with a separate negative water control. Universal barcoded forward primers were created according to Caporaso et al. (2011).

Egg and water column samples harvested at T-1 were PCR-amplified using the following conditions: 10 mL of 2.5X Quantabio 5 Prime hot start master mix (Beverly, MA, USA), 200 nM forward barcoded primer (515f; AATGATACGGCG ACCACCGAGATCTACACGCTxxxxxxxxxxxxTATGGTA ATTGTGTGYCAGCMGCCGCGGTAA, with the x region representing the bar code), 200 nM reverse primer (806r; CAAGCAGAAGACGGCATACGAGATAGTCAGCCAGCCG GACTACNVGGGTWTCTAAT), 325 ng of gDNA template, and PCR-grade dH_2_O to bring the total volume up to 25 μL. Settings for the thermal cycler (Thermo Scientific, Waltham, MA, USA) were programmed following the Earth Microbiome Project standards (Thompson et al., 2017): initial denaturation at 94°C for 2 min; 35 cycles of denaturation at 94°C for 45 sec, annealing at 50°C for 1 min, and elongation at 68°C for 30 sec; final elongation at 68°C for 10 min.

Remaining tissue and water column samples harvested from T0 to T3 (after the animals hatched) were PCR-amplified using the following hi-fidelity conditions: 12.5 μL of 2X Q5 Master Mix (New England Labs, Ipswich, MA, USA), 500 nM each of forward universal barcoded primer and reverse primer as noted for the T-1 samples, a variable amount of gDNA template dependent upon primer optimization for each tissue type (Table S1), and PCR-grade dH_2_O to bring the total volume up to 25 μL.

### Gel extraction

Samples, PCR-amplified in triplicate tubes, were pooled together prior to visualization on a 1% agarose gel. Due to the presence of contaminating host 18S rRNA DNA, bands associated with V4 region amplicons were manually gel extracted and purified using the Qiagen Gel Extraction kit per the manufacturer’s instructions with the following alterations: elution with 50 μL dH_2_O and incubation at 72°C for 5 min prior to elution.

### Sequencing and bioinformatics

Gel-purified samples were quantified *via* Qubit fluorometry (Invitrogen, Waltham, MA, USA) prior to sequencing using the Illumina 250-bp paired-end MiSeq platform (V2 500 cycle) at a final concentration of 14 pM and 16 pM for runs 1 and 2, respectively. Two MiSeq runs were used and set up by combining half of the samples from each diet into separate pools to be sequenced individually. PhiX was also spiked in the MiSeq runs at a concentration of 20% and 47%, for runs 1 and 2 respectively, for quality control. Bioinformatics-based microbiome analysis was accomplished using Quantitative Insights Into Microbial Ecology (QIIME2, v. 2020.2.0) (Bolyen et al., 2019). All reads associated with the tissue samples (i.e., both eggs and fish tissues) were denoised using DADA2 (Callahan et al., 2016) including parameters to retain the full 250 bp forward reads while trimming the reverse reads starting at 105 bp. The reverse read trimming was done after visual inspection of the reads indicated a drastic reduction in read quality before 105 bp (median quality scores ~ 2) in comparison to after 105 bp (median quality scores > 30). Forward and reverse reads were merged during DADA2 denoising, which included chimera removal on an individual sample basis (default parameter for DADA2, QIIME2 plugin). The resulting amplicon sequence variants (ASVs) were further filtered to remove low frequency ASVs [less than 0.001% of the total reads, similar to previous approaches (Bokulich et al., 2013; Xue et al., 2018; Prodan et al., 2020)], and to remove host DNA (e.g., mitochondrial and chloroplast). Taxonomy was assigned using a classifier specific to the 16S rRNA gene V4 region from the frequently-updated and robust SILVA database version 138 (Quast et al., 2013; Yilmaz et al., 2014; Glöckner et al., 2017). Two samples with very low sequence counts (< 30 reads) were removed from further analysis, one from the unprocessed egg group and another sample from the diet C intestinal swab group (Table S2), to prevent relative abundance biases. Filtered ASVs were then used to create taxonomic figures by collapsing the ASVs to shared Phylum or Family levels. Individual ASVs were used for alpha and beta diversity analyses following rarefaction to 1,297 reads per sample (one sample from the diet A intestinal swab group associated with less than 250 reads was removed during rarefaction). Differentially abundant bacterial families were identified using Analysis of Compositions of Microbiomes with Bias Correction (ANCOM-BC) (Lin and Das Peddada, 2020) within R v.4.1.0 (R Core Team, 2019).

Taxonomic figures were created in R using several packages including qiime2R v.0.99.6 (Bisanz, 2018), phyloseq v.1.27.6 (McMurdie and Holmes, 2013), vegan v.2.5–7 (Oksanen et al., 2019, ggplot2 v.3.3.5 (Wickham, 2016), complexheatmap v.2.9.1 (Gu et al., 2016). Alpha diversities were calculated using the Shannon metric (measurement of the overall diversity with relative abundance factored in), evenness metric (determination of the relative dominance by ASVs), and observed ASVs (total number of ASVs present after filtering for each group) generated *via* QIIME2. Beta diversity non-metric multidimensional scaling (NMDS) visualization plots were created using inter-group microbiome dissimilarities calculated by the unweighted UniFrac (phylogeny-based beta diversity metric without relative abundances) and weighted UniFrac (phylogeny-based beta diversity metric including relative abundances) metrics, respectively within the phyloseq package (McMurdie and Holmes, 2013) in R.

### Statistical analyses

Fish production data was analyzed using the vegan package in R. Analyses included Student’s T-test for biometrics measured at the final timepoint, and the parametric one-way analysis of variance (ANOVA) for the production period data, following confirmation of variance homogeneity *via* Levene’s test (P>0.05). The Tukey’s *post-hoc* was implemented if the ANOVA test identified significant results (P<0.05).

Alpha diversities were compared using the non-parametric one-way Kruskal-Wallis test, and statistically significantly different results were further identified using Dunn’s *post-hoc* test (P<0.05 considered significant). UniFrac-based beta diversities were compared using permutational multivariate analysis of variance (PERMANOVA with 999 permutations) with pairwiseAdonis v.0.0.1 (Arbizu, 2017) R package for *post-hoc* tests.

## Results

### Fish production data

Fish survival for all diets was greater than 98% and the introduction of probiotics didn’t significantly impact the survival rate of steelhead trout (Table 1). The system was able to provide a healthy water quality environment for the fish for the duration of the study.

On a per-tank basis, all four treatment groups (A to D) entered the linear phase of growth around the same period of time (day 128) and followed a similar growth curve pattern (Figure S1). Throughout the course of the trial, fish fed diet D had a consistently higher growth rate. This is in contrast to the lowest growth rates observed in fish fed diet C. However, following a one-way analysis of variance (ANOVA) of the overall fish weights, no group was significantly higher nor lower in average fish weight (P>0.05; Figure S1), on a per-tank basis.

As a measure of overall feed efficiency, the bi-weekly feed conversion ratio (FCR) remained under 1 for the majority of the production period (Figure S2). The only statistical difference in FCR, calculated by ANOVA on a per-tank basis, was between fish fed diets D and C observed at day 115 (P<0.05). In regard to the overall FCR, each group was highly similar, and all values were below one (Table 1). These FCR values demonstrated an appropriate rate of fish feeding, indicating that the fish were not overfed.

### Fish physiology

Individual animal biometric data (n=32 fish) comparisons were statistically assessed using multiple pairwise comparisons between fish fed diets A through D. Fish fed diet D (probiotic then commercial feed) achieved a significantly higher (P<0.05) weight than diet C fish (commercial feed then probiotic) (Table 2), on an individual basis. Generally, fish in the diet D group trended higher than those fed other diets regarding weight, length, fillet, and ribless fillet yields. Further, the ribless fillet yield of diet D fish was ~13% higher (P<0.05) than that of diet A fish (continually-fed commercial feed). The results also indicated the Fulton’s condition factor of the individual fish fed diet D were trending higher than fish fed diet A, adding confidence to the difference in fillet yields.

Apart from muscle ratio, diet C fish generally exhibited poorer biometric conditions than the fish fed other diets on an individual basis (Table 2). The VSI, which is a broad measure of energy retention in the visceral tissues, was significantly lower (P<0.05) in these fish compared to diet A fish. The lower VSI compounded with the lower growth implies the inability of the diet C fish to store enough energy to supply sufficient growth. Moreover, the Fulton’s condition factor for fish fed diet C was significantly lower than that of fish fed diet B.

### Probiotic ingestion

Fish fed diet B (continually-fed probiotic) consistently harbored the highest concentration of probiotic as measured by qPCR analysis of tissue homogenates across all of the time points sampled (T1 to T3; day 29 to day 184; Table 3). The second highest ingested concentration of probiotic was measured in fish fed diet C (commercial feed then probiotic) at T2 (day 86) and T3. In comparison, the fish fed diet A (continually-fed commercial feed) harbored no probiotic organisms detectable by qPCR until T1 (day 29). At day 86, while detectable, the amount of probiotic in fish fed either diet A or D (probiotic then commercial feed) is several orders of magnitude lower than diet B and C groups actively being fed the probiotic. The fact that the probiotic was detectable in animals never fed the probiotic (diet A) may indicate possible uptake of low levels of surviving probiotic microbes and/or spores, below the limit of detection, from the environment (e.g., originating from the air, water, equipment, etc.). This is further supported by observations that the probiotic was below qPCR-detectable levels in the water column from day 31 to 125.

### Taxonomic identification of the bacterial microbiome

**FIGURE 2:**
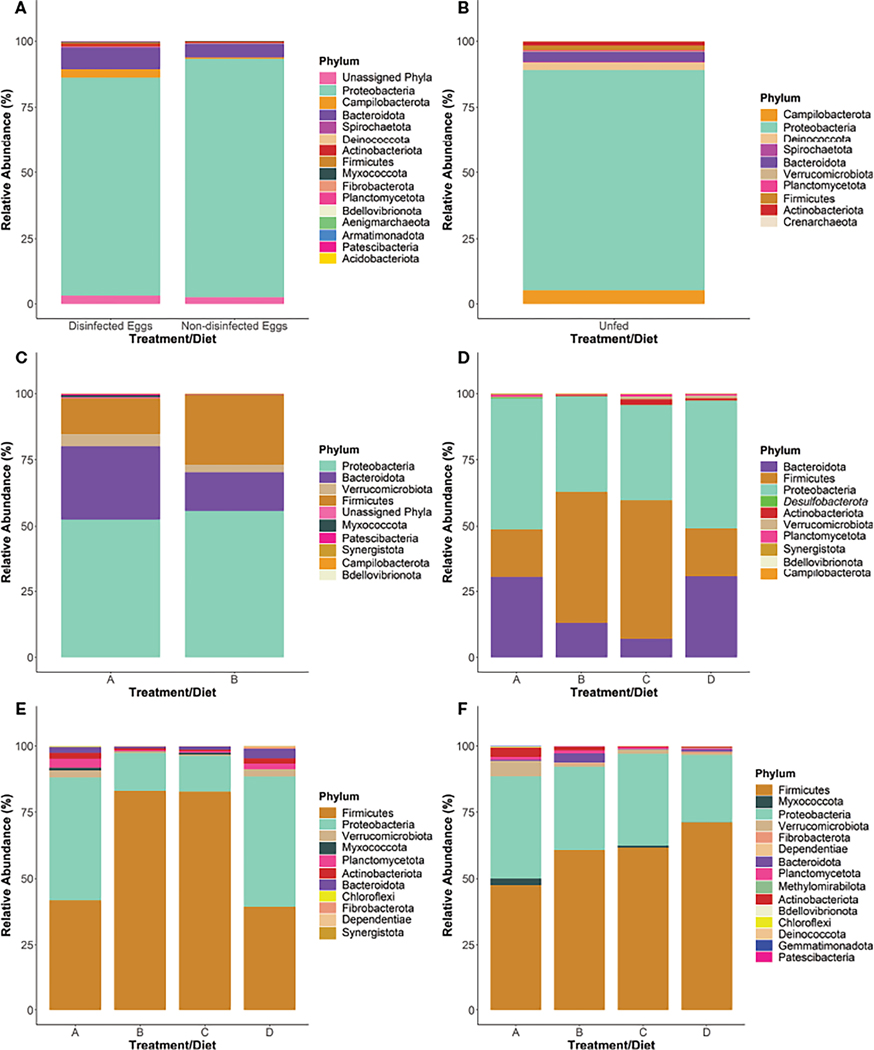
Phylum-level taxonomy of the internal bacterial microbiome of steelhead trout throughout a probiotic feeding regime. The 16S rRNA gene V4 region was sequenced at various points throughout the early lifecycle of steelhead trout at five timepoints where the total intestinal microbiome was examined: (**A**) T-1, (**B**) T0, (**C**) T1, (**D**) T2, (**E**) T3 intestinal homogenate samples. The adherent intestinal microbiome was analyzed at one time point: (**F**) T3 intestinal swab samples. Plots represent the top phyla comprising at least 90% of the total microbiome for all treatment groups. Bars were constructed using the average reads from samples specified as: five non-disinfected eggs, six disinfected eggs, 10 T0 internal homogenates, 10 T1 internal homogenates, eight T2 intestinal homogenates, 12 T3 intestinal homogenates, 10 T3 intestinal swabs for diets A, B, and D, and nine T3 intestinal swabs for diet C.

**FIGURE 3:**
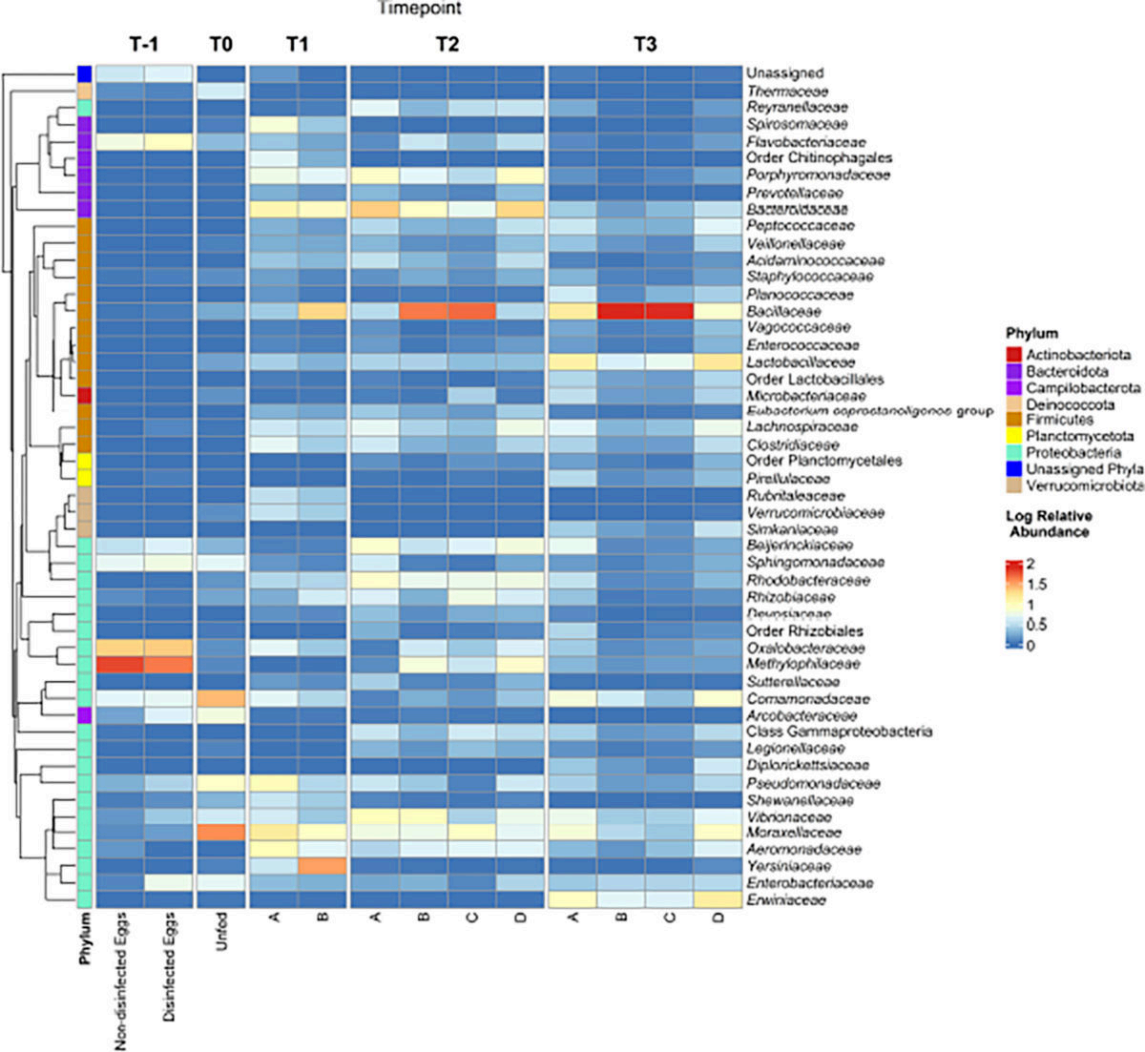
Family-level taxonomy of the internal bacterial microbiome of steelhead trout throughout a probiotic feeding regime. Following amplification of the 16S rRNA gene V4 region from fish internal GI samples collected at various timepoints during the early lifecycle of steelhead trout and fed one of four different diets (A, B, C, or D) or unfed eggs, the amplicons were sequenced *via* Illumina MiSeq. The log relative abundance of family-level taxa is plotted here. ASVs without family-level assignment are designated the higher classification as necessary. A QIIME2-generated phylogenetic tree was used to denote the phylogeny and phylum to which each family-level taxon belongs.

Sequencing generated a total of 10,071,326 and 7,999,706 reads for runs 1 and 2, respectively and bioinformatics analysis resulted in 1,381 unique ASVs. The bacterial microbiome was analyzed at the phylum (Figure 2), family (Figure 3) and genus taxon levels (Table S3). The steelhead trout intestinal microbiome is mostly comprised of Proteobacteria during early development (T-1 to T1; day −19 to day 29) (Figures 2A–C). Furthermore, the effect of in-lab iodine-treatment on the exterior of the eggs did not dramatically alter the phylum-level microbiome structure of the eggs indicating the majority of recovered ASVs at this timepoint originated from internal tissues. However, there is noticeable variation between the first three timepoints within the family-level phylogeny (Figure 3). Initially dominated by *Methylophilaceae* and *Oxalobacteraceae* at the egg stage (T-1), the fish become dominated by *Moraxellaceae*, *Comamonadaceae*, and *Pseduomonadaceae* by the first feeding at T0 (day 0). *Acinetobacter* sp. (Table S3) represented the most dominant genus at T0 within *Moraxellaceae* and they continued to represent a major genus of the internal microbiome of the fish through T1 (Table S3). By T1, the fish were no longer dominated by just a few families and begin to exhibit diversified microbiomes. For example, there were more families associated with the phylum Bacteroidota in higher relative abundance at T1 than at T0 (e.g., *Bacteroidaceae*, *Porphyromonadaceae*, and *Spirosomaceae*; Figure 3). Additionally, more families within Proteobacteria comprised a greater percentage of the microbiome from T0 to T1 including two families associated with fish pathogens (*Yersiniaceae* and *Aeromonadaceae*) (Figure 3). Fish fed the probiotic (diet B) from T0 to T1 still contained a relatively large proportion of *Acinetobacter* sp. (within *Moraxellaceae*; Figure 3) with ~5% relative abundance at T1 (Table S3). However, they had a much higher proportion of *Yersinia* sp. (within *Yersiniaceae*; Figure 3) with ~31% relative abundance (Table S3) and *Bacillus* sp. (within *Bacillaceae*; Figure 3) with ~19% relative abundance (Table S3) than diet A control-fed fish with ~2% and <1% relative abundances of *Yersinia* sp. and *Bacillus* sp., respectively (Table S3).

Between T1 and T2 (day 86), the phylum-level taxonomic structure appeared similar to T1 with Proteobacteria, Firmicutes, and Bacteroidota representing the three main phyla (Figures 2C–D). The dietary effect on the Firmicutes’ relative abundance was more exaggerated by T2. For instance, fish fed diets A and D harbored a lower proportion of Firmicutes than fish being fed probiotic diets B and C. The dominant Firmicutes families at T2 were *Lachnospiraceae* (with the most dominant organism, *Oribacterium* sp., at <3% of the total microbiome; Table S3) for fish fed diets A or D. For fish fed diets B or C, the dominant Firmicutes family was *Bacillaceae* (with the most dominant organism, *Bacillus* sp. at 41–46% of the total microbiome (Table S3; Figure 3). At T2, diet A- and D-fed fish harbored higher numbers of *Bacteroidaceae* (represented by *Bacteroides* sp.; Table S3) in their internal microbiomes compared to T1 (Figure 3). Importantly, the relative abundance of *Bacteroidaceae* in fish fed diets A and D was not as high as the *Bacillaceae* (represented by *Bacillus* sp.; Table S3) observed in diets B- and C-fed fish (Figure 3).

At the final timepoint (T3; day 184),when the animals and their intestinal tracts were largest, the total internal microbiomes within all sampled fish were dominated by the phyla Firmicutes and Proteobacteria (Figure 2E), comprised mostly of the families *Bacillaceae* and *Lactobacillaceae* within Firmicutes and *Erwiniaceae* within Proteobacteria, respectively (Figure 3). By T3, many families appear to be differentially represented, dependent upon administration of the probiotic. For example, probiotic administration using *Bacillus subtilis* correlates to the increased presence of its associated taxonomic family, *Bacillaceae*, in fish fed diets B and C compared to A and D (Figure 3). In contrast, the family *Lactobacillaceae* appeared at higher relative abundances within fish fed diets A and D compared to fish fed diets B and C. Similar to the relative abundance pattern observed with the Firmicutes-associated family *Lactobacillaceae*, Proteobacteria-associated families including *Erwiniaceae*, *Moraxaellaceae*, and *Comamonadaceae* appeared at higher levels within fish fed diets A and D compared to fish fed diets B and C (Figure 3).

Interestingly, the adherent microbiomes (i.e., swabbed intestinal samples) between fish fed all four diets appeared fairly similar (Figure 2F). However, the adherent microbiome of fish fed diet D harbored a higher proportion of Firmicutes, represented mostly by *Bacillus* sp. (Table S3), than fish fed the other three diets (Figure 2F). The second highest phylum in the adherent microbiomes was Proteobacteria, represented by the *Enterobacteriaceae* family (Table S3).

The ANCOM results, measuring the ASV abundance differential between diet groups, for the T3 intestinal homogenates indicated the following families were enriched (P<0.05) in fish fed diets A and D compared to fish fed diets B and C: *Lachnospiraceae, Clostridiaceae, Microbacteriaceae*, and *Lactobacillaceae*. Conversely, the family *Bacillaceae* was highly enriched (P<0.001) in fish fed diets B and C compared to diets A and D. No significant shifts in differential abundance were detected for the mucosal microbiomes collected *via* sterile swabs.

### Bacterial microbiome community diversity analysis

Diet A-fed fish exhibited their highest overall alpha diversities, reflecting the number and relative representation of different ASVs in a sample, at T1 (day 29) versus later timepoints, as calculated by the Shannon index (H; 5.64) and 156 observed ASVs (Table 4). Following T1, a decline in H (5.05 by T3) and observed ASVs (87 by T3) was observed for the animals fed diet A. A similar trend was identified for fish fed diet D from T2 (day 86) to T3 (day 184) with a decreasing trend in H (5.27 to 5.22) and observed ASVs (119 to 90.6) (Table 4).

Fish fed diets B or C also exhibited decreasing trends in H and observed ASVs as identified in fish fed diets A or D from T1 to T3. However, H was significantly lower in these fish than fish fed diets A or D (Table 4). In fact, the Shannon diversities of fish fed diets B and C at later time points were lower than that of the egg samples collected at the beginning of the study. The number of observed ASVs for diet C-fed fish were statistically similar to the other three groups at T2 and T3, albeit the values were trending lower than fish fed diets A or D.

One distinction for the alpha diversity results between the four diets was observed in the evenness metric, a relative measure for the representation of individual taxa (i.e., microbiomes dominated by a few taxa will have lower evenness values than microbiomes harboring taxa with similar relative abundances). Between T2 and T3, the evenness results increased 0.752 to 0.780 for fish fed diet A and 0.766 to 0.808 for fish fed diet D. On the other hand, a decrease in evenness from T2 to T3 was observed in fish fed diets B (0.517 to 0.334) or C (0.463 to 0.374).

All calculated alpha diversities of the intestinal mucosa at T3, sampled using sterile swabs, were similar between the four diets (Table 4). Notably, the overall alpha diversities (H, observed ASVs, and evenness) were lower in the mucosal microbiome compared to the total intestinal homogenate microbiome for fish fed diets A and D. This observed decrease in alpha diversities did not appear to be mirrored in fish fed diets B and C.

**FIGURE 4:**
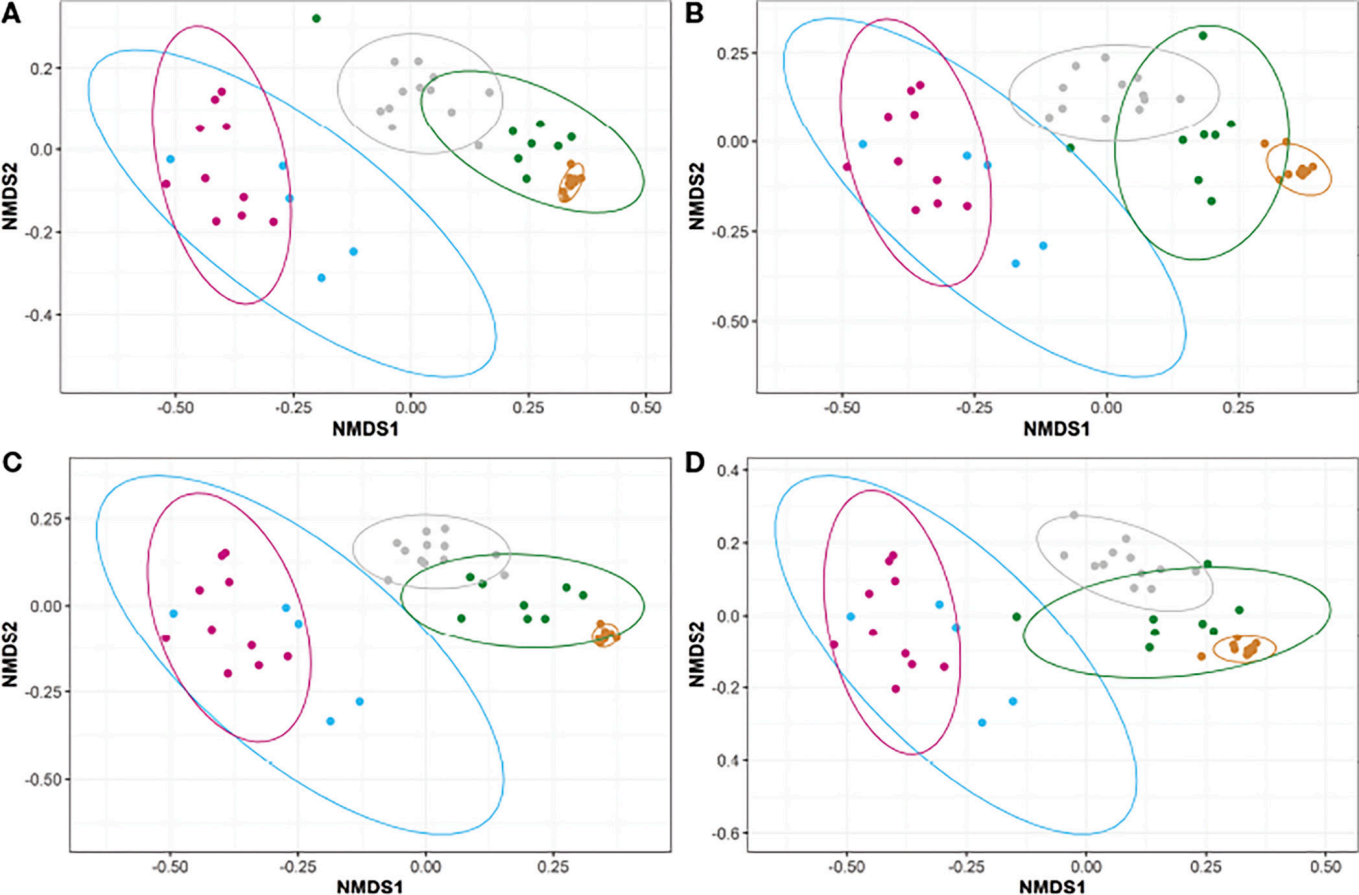
Non-metric Multidimensional Scaling (NMDS) plots representing the unweighted beta diversities of steelhead trout bacterial microbiomes for the duration of a probiotic feeding regime. Distances were calculated by unweighted UniFrac between the five timepoints for fish fed diets (**A**) A, (**B**) B, (**C**) C, and (**D**) D. Timepoints are represented by the colors cyan (T-1), maroon (T0), orange (T1), green (T2), and gray (T3). Ellipses represent the 95% confidence intervals calculated *via* Student’s T test for five non-disinfected eggs, 10 T0 internal homogenates, 10 T1 internal homogenates, eight T2 intestinal homogenates, and 12 T3 intestinal homogenates.

Beta diversities were calculated for each diet across the five timepoints, excluding the intestinal swabs, using the unweighted UniFrac method (Figure 4). Prior to initial feeding, the unweighted UniFrac beta diversities of unfed fish (the original animals shared by all treatments and collected at T-1 and T0) indicated highly similar microbiomes following a PERMANOVA pairwise test (P ~1, Figures 4A–C). However, the unweighted UniFrac results between the later timepoints (i.e., T1, T2, and T3) showed a high degree of dissimilarity (P<0.05) for all diets (Figure 4). Further, the microbiomes between unfed timepoints (i.e., T-1 and T0) and fed timepoints (i.e., T1, T2, and T3) were highly dissimilar (P<0.05) for all diets. Regardless of diet, the unweighted microbiomes appear to transition from T1 to T2 to T3 (i.e., T1 clusters closer to T2 than T3 and T2 clusters closely to both T1 and T3).

**FIGURE 5:**
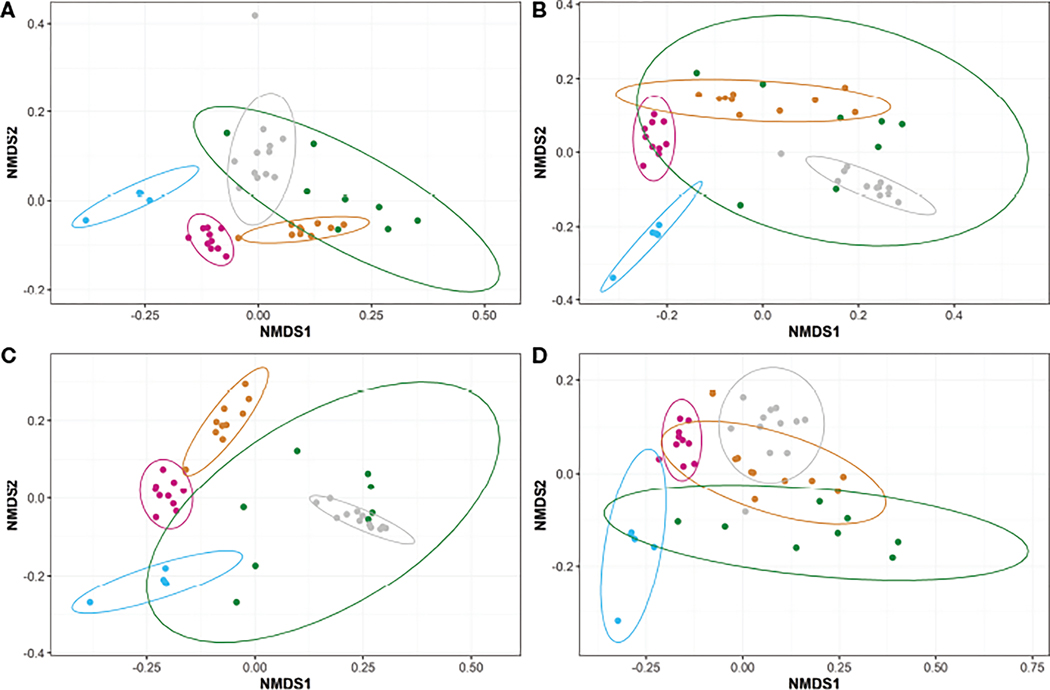
Non-metric Multidimensional Scaling (NMDS) plots representing the weighted beta diversities of steelhead trout bacterial microbiomes for the duration of a probiotic feeding regime. Distances were calculated by weighted UniFrac between the five timepoints for fish fed diets (**A**) A, (**B**) B, (**C**) C, and (**D**) D. Timepoints are represented by the colors cyan (T-1), maroon (T0), orange (T1), green (T2), and gray (T3). Ellipses represent the 95% confidence intervals calculated *via* Student’s T test for five non-disinfected eggs, 10 T0 internal homogenates, 10 T1 internal homogenates, eight T2 intestinal homogenates, and 12 T3 intestinal homogenates.

**FIGURE 6:**
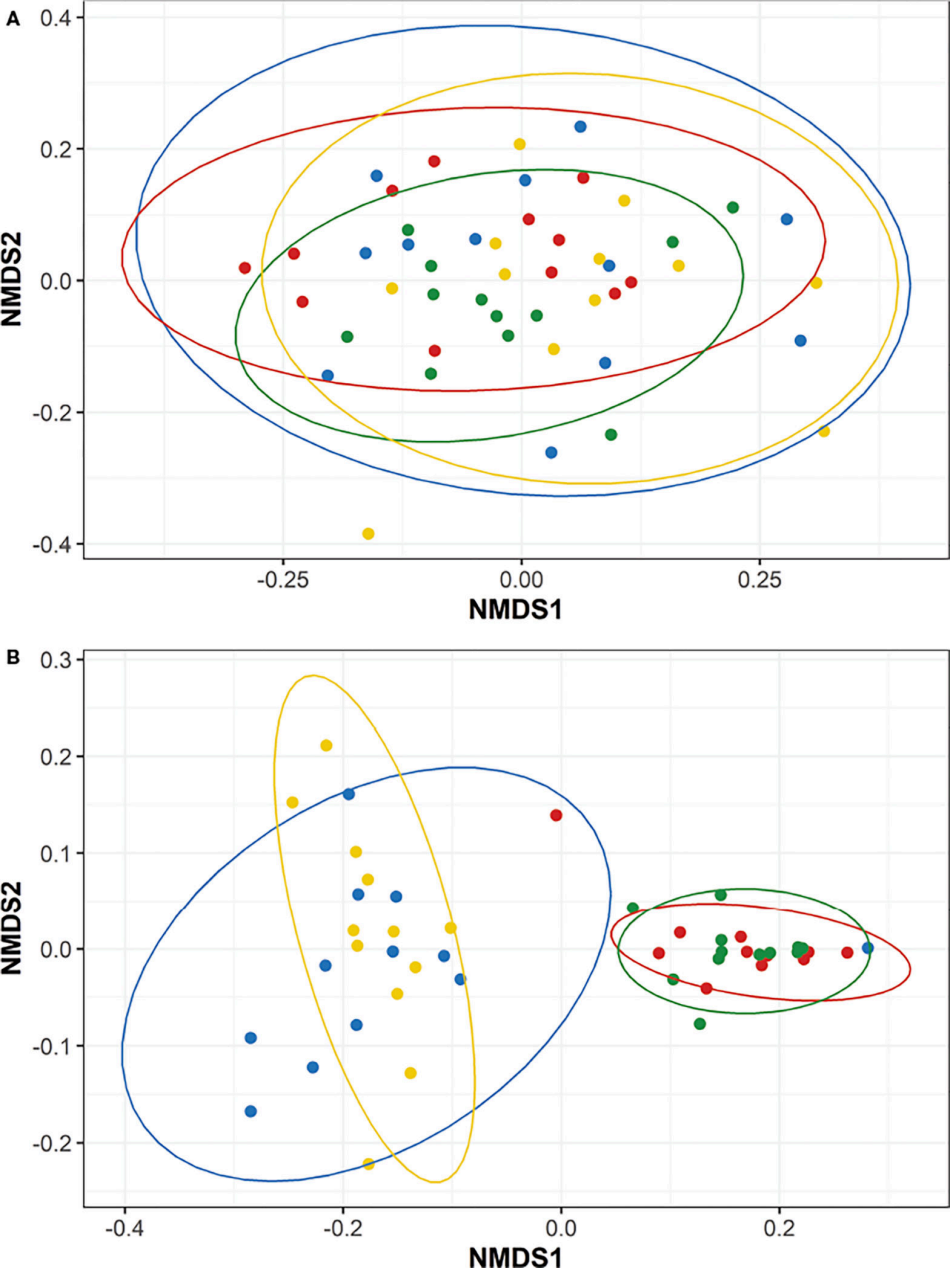
Non-metric Multidimensional Scaling (NMDS) plots representing the T3 beta diversities between steelhead trout fed different concentrations of a probiotic. Distances were calculated by (**A**) unweighted UniFrac and (**B**) weighted UniFrac between four diets: A (colored blue), B (colored red), C (colored green), and (colored yellow). Ellipses represent the 95% confidence intervals calculated *via* Student’s T test for 12 T3 intestinal homogenates.

Weighted UniFrac-calculated beta diversities, which factor in relative abundance to compare the overall trends between microbiomes, indicated the microbiomes within fish fed diets A or C were distinct between each timepoint (P<0.05; Figures 5A, C). Fish fed diets B or D also had distinct microbiomes over the course of the study with the exception between T1 to T2 (P>0.05; Figures 5B, D), wherein the microbiomes at T1 and T2 were significantly similar for fish fed diets B or D. Fish fed diet D also appeared to have more similarities between their microbiomes present at T1, the timepoint exhibiting the highest overall diversities (Table 4), and T3 (Figure 5D). Intra-timepoint dietary effects were also analyzed for beta diversity differences. All diets/treatments exhibited a high degree of similarity (P>0.05) for the unweighted UniFrac metrics (Figure S3 and Figure 6A) at each timepoint. The weighted microbiomes, however, indicated distinct microbiomes (P<0.05) present at T1 (between groups A and B; Figure S3) and T2 (between groups A and C; Figure S3). However, the intestinal homogenates’ microbiomes clustered into two groups (P<0.05) with regard to the probiotic feeding regime in place by the end of the study at T3. In other words, the microbiomes of fish fed diets A or D were highly similar, and the microbiomes of fish fed diets B or C were highly similar (Figure 6B). Comparison between fish fed diets A or D and fish fed diets B or C indicated a clear distinction between the two clustered groups.

## Discussion

Probiotic use in aquaculture has been implemented to provide several key benefits to cultured fish. One probiotic organism in particular, *Bacillus subtilis*, has been extensively studied for its impact on aquatic host growth, immune function, microbiome modulation, and pathogen protection among other benefits (Kumar et al., 2008; Merrifield et al., 2010; Standen et al., 2015; Cheng et al., 2019). The findings of this study demonstrated that exposure to a *B. subtilis* probiotic exclusively during the initial stages of intestinal development (diet D) benefitted the fish significantly better than feeding probiotic at a later stage of growth (diet C). In fact, fish fed probiotic only during the later developmental stages tended to perform worse than all the other treatment groups. The poorer performance of diet C fish may be linked to their lower VSI (Table 3) in comparison to fish not fed any probiotic (diet A) (Adhami et al., 2021; Liu et al., 2021). There is evidence that probiotic administration may actually diminish the health of fish under some growth conditions (Ramos et al., 2017). However, continued delivery of the probiotic without cessation (diet B) in this study did not appear to negatively impact fish growth compared to fish fed no probiotic (diet A), nor did it benefit the fish any more than limited early exposure to the probiotic (diet D) in regard to the fish biometrics. The level of probiotic administered and the pre-existing microbiome likely influenced the observed outcomes.

*Bacillus subtilis* probiotic ingestion by fish through their feed was confirmed *via* qPCR analysis (Table 3). The amount of *B. subtilis* present in fish groups B and C was similar, suggesting near equal feeding and consumption. Taxonomic analyses also agree with the qPCR findings by indicating a large proportion of the diet B- and C-fed fish bacterial microbiomes were dominated by *Bacillaceae*, the family with which *B. subtilis* is associated (Figure 3). Curiously, the *Bacillus subtilis* probiotic was detected *via* qPCR in the control fish (diet A) by day 29 (T1) and in both diet A- and D-fed fish at days 86 (T2) and 184 (T3) (Table 3). Further, *Bacillaceae* was present in the microbiome analysis of diet A- and D-fed fish at all timepoints, albeit at a much lower relative abundance than diets B- and C-fed fish (Figure 3). The source of *Bacillus* sp. in the cases where the fish weren’t actively being fed probiotic could have originated from the commercial feed (Wilkes Walburn et al., 2019) or *via* cross contamination from the water column at a level below our limit of detection for qPCR analysis (10^2^ CFU/g).

Though diet D-fed fish had significantly increased fish growth over the other three groups on an individual basis, the total microbiome (i.e., feces + mucosa) of these animals was similar (P>0.05; Figure 6, Figure S3) to fish never exposed to probiotic (diet A). In contrast, continued administration of the probiotic after T1 (diets B and C) led to a reduction in the number of observed ASVs compared to fish fed diets A and D (Table 4), and it also led to dominance by much fewer taxa (including a majority by *Bacillus* sp.; Table S3) thus reducing Shannon diversities. In addition to the higher alpha diversities, fish fed diets A and D exhibited an increased relative abundance of other bacterial families, notably *Lactobacillaceae* (Figure 3), compared to fish fed diets B and C. Several bacteria in the *Lactobacillaceae* family have been isolated from trout and identified as potential probiotics (Chapagain et al., 2019; Mohammadian et al., 2019; Soltani et al., 2019), including *Lactobacillus plantarum* that has been used as effective immune-supporting probiotics in trout cultures (Balcázar et al., 2007; Balcázar et al., 2008; Vendrell et al., 2008). Thus, the increased microbiome diversity in the diet A and D fish may have afforded benefits to the animals.

In comparison to the microbiome in the total intestinal homogenates, the mucosal-only based microbiome (collected *via* swabs) showed a high degree of similarity between all four diets indicating no significant impact of the probiotic treatments on the adherent microbiomes. Though statistically similar, diet D-fed fish had the highest levels of Firmicutes (Figure 2F), which was dominated by *Bacillus* sp. (Table S3), compared to the other three diets, suggesting some level of colonization by the probiotic. Thus, the mucosal colonization by the probiotic may be more effective when exposed to the fish earlier in their development (diet D), compared to fish consistently exposed to the probiotic or fed later in development (diets B and C, respectively). Additionally, the majority of bacterial families shed in the feces (total microbiome minus mucosal microbiome) of fish fed diets A or D were not classified as *Bacillaceae*; transient bacteria shed in the feces of diet A- and D-fed fish were much more diverse compared to the fish fed diets B and C.

Because the diet-based separation of microbiomes, as examined through weighted beta diversity analysis, was not as apparent until day 184 (Figures 6 and S3), host age may also be an important factor in microbiome development in addition to diet. Temporal shifts in the microbiome communities occurred as the fish matured, illustrated by a trend from low diversity microbiomes (Table 4) dominated primarily by Proteobacteria (Figure 2), specifically *Methylophilaceae* (Figure 3), at the egg stage towards a high diversity microbiome dominated by Proteobacteria, Firmicutes, and Bacteroidota at T1 (day 29). These three phyla likely make up a portion of the core microbiome of several different fish species including trout (Tarnecki et al., 2017; Hines, 2022). The initial increasing shifts in microbiome constituency from unfed (T-1) to fed (T1) fish ended at T1 as illustrated by the alpha diversities (Table 4). Microbiome constituencies then appear to transition from T1 to T2 to T3 in a more ordered and overlapping fashion for all four diets (Figure 4). This slower change in the bacterial community suggests some establishment by persistent ASVs following the peak overall diversity at day 29. Interestingly, fish fed diet D appear to harbor microbiomes at T3 more similar to T1 than the other dietary groups. This is reflected in the overall highest Shannon diversity present at T3 compared to the other groups of fish. Thus, the early exposure of the probiotic may have lasting benefits that aid in maintaining high diversity microbiomes that aren’t dominated by just a few taxa.

There are limits to using 16S rRNA gene sequences for absolute taxonomic findings, including the length of the primer, primer mismatches and the number of cycles used for PCR (Piñol et al., 2015; Sze and Schloss, 2019; Witzke et al., 2020). Nevertheless relative taxonomic findings can yield important insights into the composition of the microbiome. To build off of this study, multi-omics approaches such as transcriptomics, metagenomics, and metabolomics (Rasmussen et al., 2022; Rebollar et al., 2016) would help to investigate the physiological functions and mechanisms of microbiome members and help guide the development of more effective probiotic regimens for aquacultural practices.

## Conclusions

In conclusion, while the amount of research surrounding probiotic efficacy in fish feed is extensive, the efficacy of timed probiotic exposure is less well understood. In the present study, different treatment groups were defined by probiotic administration that was varied with respect to fish development, starting with eggs. Temporal shifts in the bacterial microbiome were observed, with the highest level of microbiome community diversity present after 29 days across all diets. The study results suggest implementation of probiotic only during initial intestinal development will increase the overall growth of the fish, increase overall microbial diversities within the intestinal tract, and possibly allow for better establishment of the probiotic within the adherent microbiome. There was a significant decrease in the overall diversities of fish total internal microbiomes (i.e., feces plus mucosa) when the probiotic was being administered. The adherent mucosal microbiomes differed little between the four diets, but the faster growing diet D-fed fish had the highest adherent probiotic levels. Overall, the diversity of the microbiome seems to be an important factor in driving animal production to higher levels; diet D animals with the highest growth rates had the highest microbiome alpha diversity with the most unique taxa at a fairly even distribution. The lack of major variability in the mucosal microbiome indicates a possible set of resilient bacteria that constitute the core microbiome for steelhead trout (Roeselers et al., 2011; Wong et al., 2013; Lyons et al., 2017; Tarnecki et al., 2017). Overall, these results indicate feeding trout a *Bacillus subtilis* probiotic is most beneficial when supplied as a short exposure early in intestinal development during the early hatchery phase, rather than beginning probiotic regimens later in fish development or continuous use. This finding has important economic implications for producers and the interpretation of results from studies administering the probiotic during later development.

## Supplementary Material

Table S3

Supplementary Data

## Figures and Tables

**TABLE 1:** Production period (T2 to T3) measurements of steelhead trout following probiotic treatment.

Diet	Initial sample size (n)	Survival (%)^1^	FCR^2^
**A (continually-fed commercial)**	206	98.7	0.937 ± 0.046
**B (continually-fed probiotic)**	168	99.4	0.934 ± 0.012
**C (commercial to probiotic)**	169	99.4	0.971 ± 0.028
**D (probiotic to commercial)**	191	100	0.899 ± 0.015

1 No error is presented here due to the step-wise expansion from two to four tanks during the production period; instead this represents the mean survival for each diet across the entire production period.

2 Feed conversion ratio (FCR) calculated as mean ± standard error mean on a per tank basis.

**TABLE 2:** Physiological biometric measurements of individual steelhead trout at T3 (day 184^1^).

Diet	A	B	C	D
**Sample size (n)**	32	32	32	32
**Weight (g)**	125 ± 4.97	129 ± 6.31	121 ± 6.45^a^	138 ± 5.29^b^
**Length (cm)**	21.3 ± 0.26	21.2 ± 0.31	21.1 ± 0.31	21.9 ± 0.30
**Fillet (g)**	34.4 ± 1.53	35.8 ± 1.94	34.1 ± 1.97	38.1 ± 1.59
**Ribless fillet (g)**	30.4 ± 1.35^a^	32.2 ± 1.82	30.7 ± 1.84	34.9 ± 1.52^b^
**HSI**	1.58 ± 0.06	1.71 ± 0.05	1.70 ± 0.03	1.62 ± 0.07
**VSI**	16.2 ± 0.39^a^	15.3 ± 0.42	15.0 ± 0.39^b^	16.1 ± 0.42
**Muscle ratio (%)** ^2^	54.8 ± 0.76	55.3 ± 0.76	56.1 ± 0.41	55.4 ± 1.04
**Fulton’s condition factor**	1.27 ± 0.02	1.31 ± 0.02^a^	1.24 ± 0.02^b^	1.30 ± 0.02

1 Error represented by the mean ± standard error mean.

2 Ratio of total fillet yield (both sides of the fish) to total fish weight.

ab Different superscript letters represent P<0.05 within a horizontal row after multiple pairwise comparisons using the t-test.

**TABLE 3:** Steelhead trout probiotic ingestion as detected by qPCR analysis^1^.

Sampling day						

Diet	3^3^	7^3^	11^3^	29 (T1)^4^	86 (T2)^5^	184 (T3)^6^
**A**	ND^2^	ND	ND	8.09E+01 ± 3.46E+01	2.22E+02 ± 6.05E+01	1.45E+02 ± 1.53E+01
**B**	1.51E+02 ± 8.78E+01	3.08E+03 ± 2.13E+03	8.99E+03 ± 8.64E+03	6.43E+04 ± 1.60E+04	3.88E+05 ± 9.72E+04	2.74E+05 ± 5.49E+04
**C**	N/A	N/A	N/A	N/A	3.33E+05 ± 6.08E+04	1.65E+05 ± 2.24E+04
**D**	N/A	N/A	N/A	N/A	8.32E+02 ± 7.46E+02	5.80E+02 ± 4.22E+02

1 Error represented by the mean probiotic concentration (CFU/g) ± standard error mean.

2 Not detected (ND), below the limit of detection for the qPCR analysis.

3 n = 4 fish (diets A and B).

4 n = 18 fish (diet A), 11 fish (diet B).

5 n = 18 fish (diet A) or 12 fish (diets B, C, and D).

6 n = 12 fish.

N/A means not applicable.

**TABLE 4:** Summary of alpha diverisity analyses (mean ± SEM).

Timepoint	Diet	Shannon	Evenness	Observed ASVs
**T-1**	**Non-disinfected**	2.70 ± 0.59	0.614 ± 0.12	48.2 ± 33.2
	**Disinfected**	3.26 ± 0.90	0.499 ± 0.11	43.8 ± 8.70

**T1**	**A**	5.64 ± 0.45^a^	0.775 ± 0.04^a^	156 ± 25.0^a^
	**B**	3.95 ± 1.24^b^	0.573 ± 0.16^b^	115 ± 31.5^b^

**T2**	**A**	5.05 ± 0.41^a^	0.752 ± 0.06^a^	115 ± 33.6^ac^
	**B**	3.22 ± 0.82^b^	0.517 ± 0.10^b^	75.5 ± 20.0^bc^
	**C**	2.98 ± 1.41^b^	0.463 ± 0.19^b^	83.0 ± 23.9^bc^
	**D**	5.27 ± 0.52^a^	0.766 ± 0.05^a^	119 ± 26.4^ac^

**T3-Homogenate**	**A**	5.05 ± 1.47^a^	0.780 ± 0.20^a^	87.0 ± 30.8^ac^
	**B**	1.96 ± 0.63^b^	0.334 ± 0.10^b^	60.8 ± 17.9^bc^
	**C**	2.29 ± 0.60^b^	0.374 ± 0.08^b^	69.3 ± 15.1^bc^
	**D**	5.22 ± 0.62^a^	0.808 ± 0.11^a^	90.6 ± 16.6^ac^

**T3-Swab**	**A**	2.74 ± 1.91	0.505 ± 0.29	45.0 ± 44.4
	**B**	2.36 ± 1.26	0.505 ± 0.22	25.4 ± 12.2
	**C**	2.04 ± 0.93	0.432 ± 0.16	31.1 ± 21.2
	**D**	1.43 ± 1.42	0.302 ± 0.22	24.2 ± 23.9

abc Different superscript letters represent P<0.05 between diets at a specific timepoint within a given diversity metric following one-way Kruskal Wallis with Dunn post-hoc test.

## Data Availability

The datasets presented in this study can be found in online repositories. The names of the repository/repositories and accession number(s) can be found below: https://www.ncbi.nlm.nih.gov/, Sequence Read Archive (SRA) under BioProject ID PRJNA750741.
