# Supplementary material for: Steelhead trout (Oncorhynchus mykiss) fed probiotic during the earliest developmental stages have enhanced growth rates and intestinal microbiome bacterial diversity
Source: Front Mar Sci. Author manuscript; Available in PMC 2024 Dec 11. (PMC11633451; doi:10.3389/fmars.2022.1021647)
Supplement: Supplementary Data [file NIHMS2001559-supplement-Supplementary_Data.docx]

**Supplementary Information**

**Table S1. Polymerase and template gDNA variable concentration based upon tissue type.**

| **Timepoint** | **Tissue type** | **gDNA amount (ng)** | **Polymerase** |  |
| --- | --- | --- | --- | --- |
| T-1 | Eggs and water column | 325 | Quantabio 5 Prime HotStart^1^ | |
| T0 | Headless/gill-less | 120 | NEB Q5 |  |
| T1 | Headless/gill-less | 120 | NEB Q5 |  |
| T2 | Intestinal segment | 30 | NEB Q5 |  |
| T3 | Intestinal segment | 30 | NEB Q5 |  |
| T0-T3 | Water column | 30 | NEB Q5 |  |

^1^ Insufficient gDNA template for amplification using new NEB Q5 enzyme

**Table S2. Breakdown of the number of sequenced samples.**

| **Timepoints** | **n (per treatment group)** |
| --- | --- |
| **T-1** | 6^1^ |
| **T0** | 10 |
| **T1** | 10 |
| **T2** | 8 |
| **T3 (homogenate)** | 12 |
| **T3 (swabs)** | 10^2^ |

^1^ One sample removed from the non-disinfected egg group due to a low read count following filtering (n=5)

^2^ One sample removed from diet C group and one from diet A group (for diversity analyses only) due to a low read count following filtering

(n=9)

**Table S3. ASV relative abundance table.**

See the separate Excel file

**
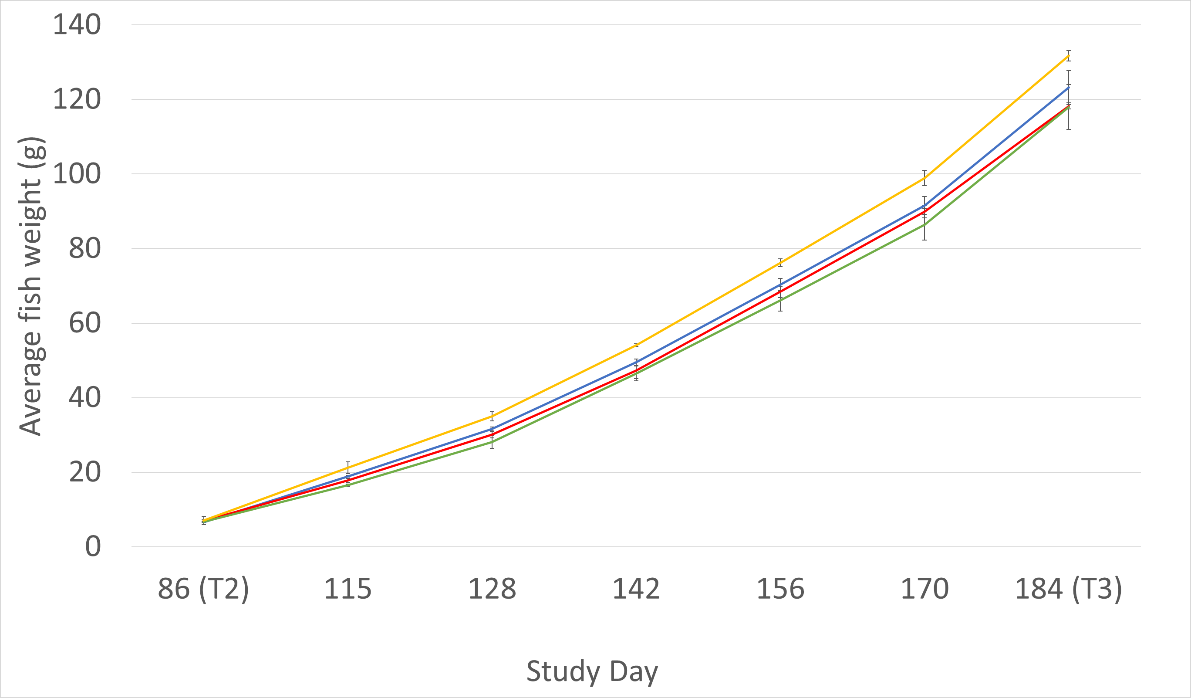
**

**Figure S1. Production period growth curve based on bi-weekly weights of steelhead trout on a per tank basis**. Each diet is represented as follows: A (blue), B (red), C (green), and D (yellow). Error bars represent the mean ± standard error mean of two tanks (days 86 and 115), three tanks (day 128), or four tanks (days 142-184).

**
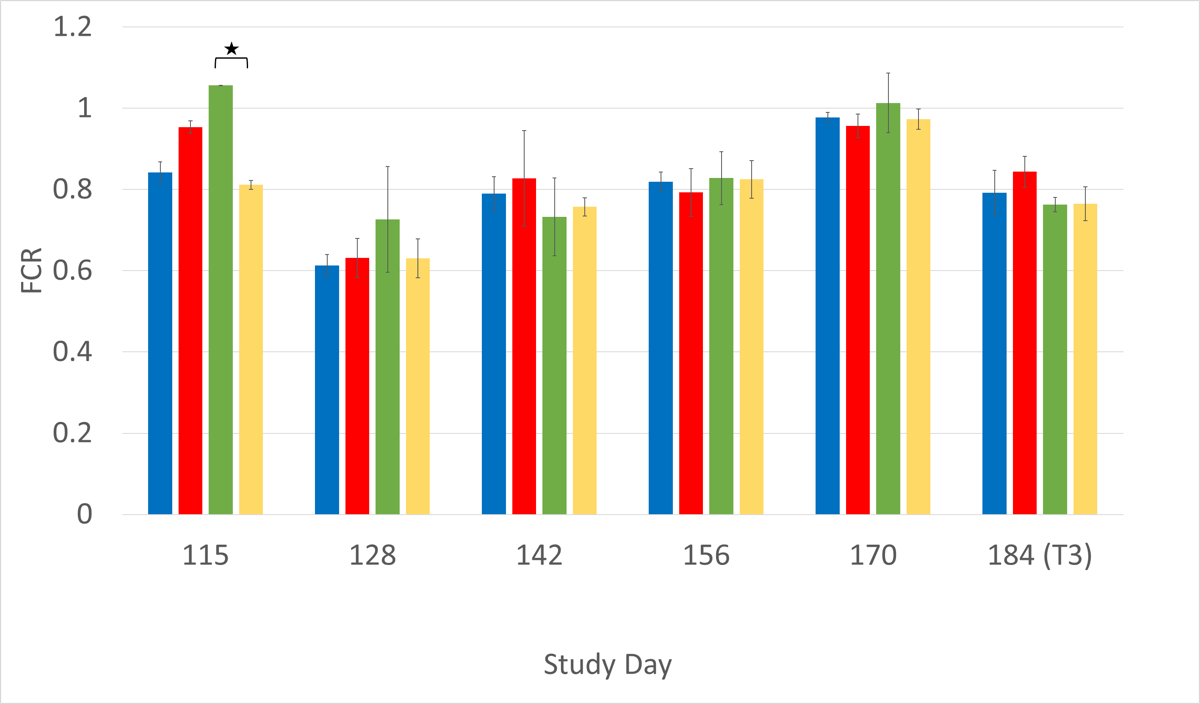
**

**Figure S2. Feed conversion ratio (FCR) during the production period of steelhead trout on a per tank basis**. Each diet is represented as follows A (blue), B (red), C (green), D (yellow). Error bars represent the mean ± standard error mean of two tanks (day 115), three tanks (day 128), or four tanks (days 142-184). One star represents P<0.05 following one-way ANOVA with Tukey’s post-hoc test.


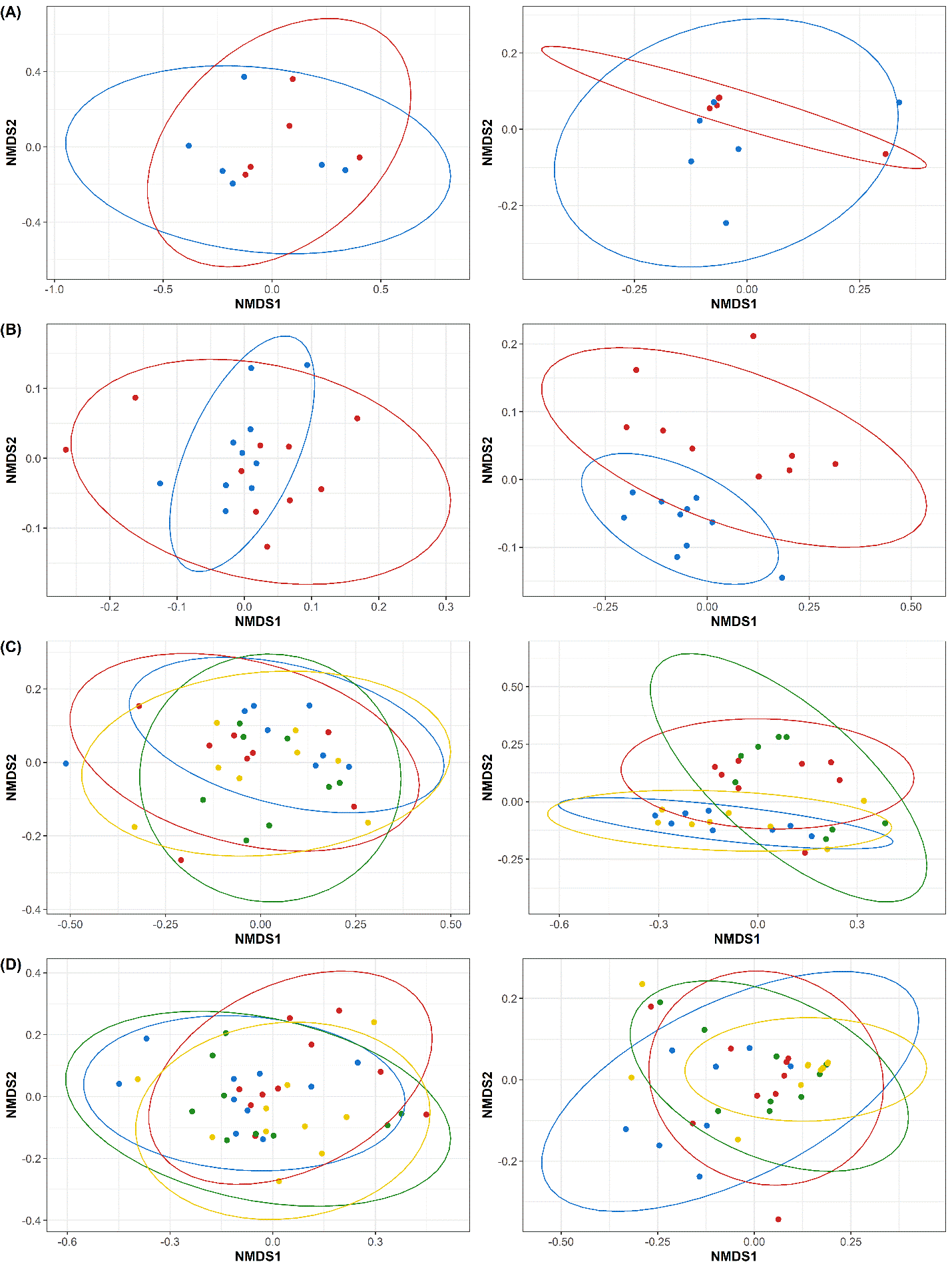


**Figure S3. Non-metric Multidimensional Scaling (NMDS) plots representing the unweighted and weighted beta diversities of steelhead trout bacterial microbiomes for the duration of a probiotic feeding regime.** Distances were calculated by (left column) unweighted and (right column) weighted UniFrac between the four diets for fish sampled at timepoints (A) T-1, (B) T1, (C) T2, and (D) T3 intestinal swabs. Colors represent the following diets/treatments: A/disinfected eggs (blue), B/non-disinfected eggs (red), C (green), and D (yellow). Ellipses represent the 95% confidence intervals calculated via Student’s T test for six disinfected eggs, five non-disinfected eggs, 10 T1 internal homogenates, eight T2 intestinal homogenates, and 10 T3 intestinal swabs (nine for diet C).
